# Quality and Authenticity Control of Fruit Juices-A Review

**DOI:** 10.3390/molecules24061014

**Published:** 2019-03-13

**Authors:** Marilena E. Dasenaki, Nikolaos S. Thomaidis

**Affiliations:** Laboratory of Analytical Chemistry, Department of Chemistry, National and Kapodistrian University of Athens, Panepistimiopolis Zographou, 15771 Athens, Greece; ntho@chem.uoa.gr

**Keywords:** fruit juice, authenticity, quality, analytical techniques

## Abstract

Food fraud, being the act of intentional adulteration of food for financial advantage, has vexed the consumers and the food industry throughout history. According to the European Committee on the Environment, Public Health and Food Safety, fruit juices are included in the top 10 food products that are most at risk of food fraud. Therefore, reliable, efficient, sensitive and cost-effective analytical methodologies need to be developed continuously to guarantee fruit juice quality and safety. This review covers the latest advances in the past ten years concerning the targeted and non-targeted methodologies that have been developed to assure fruit juice authenticity and to preclude adulteration. Emphasis is placed on the use of hyphenated techniques and on the constantly-growing role of MS-based metabolomics in fruit juice quality control area.

## 1. Introduction

The globalization of the food trade and world food markets over the last few decades have made an enormous variety of food products available to consumers and the demand for high quality food products is constantly increasing. Economically Motivated Adulteration (EMA) of food, also known as food fraud, is the intentional adulteration of food for financial advantage [1]. According to the Global Food Safety Initiative (GFSI), “food fraud is a collective term encompassing the deliberate and intentional substitution, addition, tampering or misrepresentation of food, food ingredients or food packaging, labelling, product information or false or misleading statements made about a product for economic gain that could impact consumer health” [2]. Fraudulent acts, such as the adulteration with cheaper ingredients and the false claim of origin (geographical or varietal), reduce the quality of the products, mislead the consumer and may even imply a health risk. Thus, food authenticity is a major concern for all involved in the food trade: consumers, consumer protection authorities and also producers and dealers. 

Fruit juices, and especially orange and apple juice, belong to the most targeted food commodities for adulteration and fraud [3]. According to the Association of the Industry of Juices and Nectars of the European Union (AIJN), 9.3 billion liters of fruit juices and nectars were consumed in the EU in 2016 [4]. The mounting focus of consumers toward a healthier diet including a lot of fruits and vegetables, has driven the evolution of the juice market which has been steadily growing across developing and developed countries. Apart from orange juice, which remains the most popular and widely consumed fruit juice produced in the largest volume worldwide, other fruit juice types, such as pomegranate or berry-based juices have gained a high reputation and are being sold as high-quality food items, due to their remarkable health benefits, including prevention against cancer and cardiovascular disease [5]. Taking into consideration the large fruit juice quantities consumed worldwide, the identification of juice adulteration and fraud is of great economic importance and effective control systems are utterly necessary to protect consumers from impure and fraudulently presented fruit juices [6]. 

The most frequent adulteration practices within the fruit juice industry involve dilution with water, addition of sugars, pulp wash or other additives and juice-to-juice adulteration, defined as the undeclared addition of a juice of lesser value to a product. All such forms of adulteration result in lost value for the consumer and may even jeopardize consumers’ health causing allergic reactions [7]. As a result, quality specifications and identity standards have been developed by regulatory authorities and industry to assess quality and authenticity of fruit juices. The composition of fruit juices, concentrated fruit juices, dehydrated fruit juices and fruit nectars, their reserved names, their manufacture and labelling characteristics are subject to specific Community rules under Directive 2012/12/EU, amending Council Directive 2001/112/EC. Moreover, AIJN has established the Reference Guidelines of the AIJN Code of Practices, based on pure, authentic juices, which can be used to evaluate juices with regards to quality, authenticity and identity [8]. The diversity in adulteration techniques, the natural variation of fruits of different geographical and varietal origin along with the different storage conditions and processing techniques, make the detection and prevention of juice adulteration a very complicated task. In this sense, effective, reliable and rapid food authentication methods represent a valuable and irreplaceable tool for the authorities to set up control systems to ensure juice quality and safety and to promote mitigation of fraud. 

The most frequently used analytical methods are targeted analyses which focus on the detection and identification of a particular compound or class of compounds (amino acids, organic acids, sugars, etc.). These components may serve as biomarkers of authentic products or indicate the presence of an adulterant [9]. The targeted approach provides reliable and sensitive identification and accurate quantification of specific compounds and, combined with chemometrics, can be used to determine characteristic patterns of compounds or parameters indicating the adulteration of a sample or its varietal and geographical origin [10]. However, with targeted methods, chemicals which are not selected beforehand cannot be detected, no matter how high their concentration might be in the sample. In this context, untargeted methods are becoming more and more popular as researchers have to look more widely to obtain an integrated overview of the chemical composition of the investigated sample. Untargeted methods are based on a holistic approach aiming to identify compounds from samples where the molecular content is unknown [11]. Advanced statistical tools and multivariate data analysis are used to provide the required quality and authenticity information. 

One of the most recent advanced analytical platforms is metabolomics, in which the entire composition of metabolites of a system or organism is determined and characterized. Metabolites are regarded as the final products of the genome, influenced also by its interaction with the environment, and so the metabolic fingerprint is characteristic for every species [12]. Especially for juices, the enormous diversity of plant metabolite chemical structures render metabolomics a rather challenging task. In the last few years, metabolomic studies have experienced a notable increase in interest, providing valuable information and novel analytical tools to ensure juice authenticity and quality [13,14].

The evolution and use of novel analytical methods, along with advanced statistical and mathematical techniques, to assess food authenticity has been the subject of several review articles so far [12,15,16]. However, current advancements concerning fruit juice authenticity assessment have not been recently reviewed. This review will describe the main analytical methodologies and chemometric tools used for the evaluation of fruit juice authenticity and the detection of juice adulteration in the last decade. Applications, advantages, challenges and future trends of metabolomic approaches for the authentication of juices are highlighted. 

## 2. Analytical Techniques

### 2.1. DNA-based Techniques

DNA-based technologies, mainly using PCR, real-time PCR, High Resolution Melting (HRM) analysis, microarrays and Next Generation Sequencing, have proved to be useful tools for a number of food authentication approaches [17]. They are considered more reliable compared to chemical analysis due to DNA’s stability under different environmental conditions, type of farming and production techniques [17]. DNA is considered a good authentication marker as the molecule is able to retain sequence-specific information during processing and thus DNA-based methodologies can unequivocally identify a species or variety and define if it is present in processed food [18]. DNA-based methodologies are characterized by short sample preparation, high sample throughput, good inter-laboratory reproducibility and low operating costs [19]. In spite these advantages, the number of applications in fruit juice authenticity assessment is rather limited (Table 1). 

In the case of orange juice, dual-probe real time PCR (qPCR) has been used to exploit adulteration with mandarin juice [20,21]. Aldeguer et al. used single-nucleotide polymorphism at the chloroplast chromosome trnL–trnF intergenic region to differentiate mandarin from orange DNA [20], while Pardo used two TaqMan^®^ probes to evaluate the detection of the adulteration of orange juice with mandarin [21]. The adulteration was detected down to 5% *v*/*v* and 1% *v*/*v*, respectively. PCR and Laboratory-on-a-Chip Capillary Electrophoresis (PCR-LOC) analysis was also used to detect the addition of mandarin and grapefruit juice to orange juice: a PCR restriction fragment length polymorphism (RFLP) assay was used to detect grapefruit juice down to 10% while a PCR heteroduplex assay was successfully applied to the LOC system for the detection of mandarin juice to 2.5% [22]. 

Denaturing High Performance Liquid Chromatography (DHPLC) of DNA fragments previously amplified by PCR was used to discriminate seven fruit juices, proving it to be a powerful technique that could be used for labelling regulation on fruit juice [23]. The discrimination of orange, mango, peach, pear and pineapple juices was also studied by Faria et al. through the evaluation of the effectiveness of HRM analysis. A distinct differentiation between these juices was provided, easily visualized through the melt curve difference graphs [18]. Most recently, Marieschi et al. developed a method based on Sequence-Characterized Amplified Region (SCAR) markers in order to detect juice to-juice adulteration of pomegranate juice with 10 other juices; limits of detection of up to 1% of each adulterant were achieved [19]. SCAR markers were initially developed for the detection and identification of plants and have been used since as authenticity markers for several food products [17].

### 2.2. Physicochemical Analyses

Conventionally, the authenticity of a juice product can be confirmed through the evaluation of selected physicochemical parameters, such as the total soluble solids (TSS), titratable acidity (TA), pH, L-ascorbic acid content, formol number and ash. In most cases, the methods of analysis used are the ones using refractometry, titrimetry, gravimetry and potentiometry [24] recommended by the International Federation of Fruit Juice Producers (IFU). This approach is commonly employed by quality-control labs, as it is a rapid and cost-effective way to primarily assess the authenticity of a fruit juice. Values are compared to those reported by the AIJN which have been determined in 100% pure juices and are internationally accepted by producers, users and control agencies. 

The determination of selected physicochemical parameters has been used to exploit potential adulteration or to classify juices according to their geographical and varietal origin [25,26,27,28,29,30]. Water and sugar addition to juice samples can be revealed through the evaluation of Brix value: A alue below the required specification indicates overdilution of the juice with water while a higher Brix value is indicative of the addition of sugar [25]. Significant differences in pH, TA and Brix values were reported in different varieties of pomegranate [29] and also between homemade and commercial samples [30]. Homemade pomegranate juices had a low acid content and correspondingly higher pH than commercial juices, as acidity value is known to decrease significantly during fruit ripening. Therefore, TA reduction can be used as a standard criterion for the determination of the maturity phase of fruits [30]. Additionally, the % maturity index was calculated (ratio of SSC to TA value) which is a very good indicator of fruit maturity and, consequently, of the juice taste. According maturity index, pomegranate varieties can be classified as sour, sour- sweet and sweet [29]. 

The evaluation of these physicochemical parameters can contribute to the knowledge of specific juice characteristics and, eventually, to the setting of reference guidelines. In this context, an extensive characterization of commercial Spanish lemon juices was reported by Lorente et al., through the determination of the physicochemical characteristics in 92 samples [28]. Τhe phytochemical characterization of Spanish pomegranate juices, belonging in 15 different cultivars, was performed by Mena et al. and advanced chemometrics were used to classify the samples. Principal Component Analysis (PCA) was used and the first three principal components could explain 74.3% of the total variance [31]. Nevertheless, the usual analytical techniques used for the determination of these parameters are often unable to detect small differences that could indicate low-level adulteration. The compositional differences between juices with similar characteristics (color, aroma etc.) may not be strong enough to detect the fraudulent addition of one juice to the other and juice-to juice adulteration remains undisclosed [26]. 

### 2.3. Isotope and Elemental Techniques

#### 2.3.1. Stable Isotope Ratios Analysis

Isotopic methods, based on the determination of isotope ratios (δ^2^H, δ^13^C, δ^18^O, δ^15^N, δ^34^S and δ^87^Sr), comprise some of the most efficient techniques to assess fruit juice authenticity (Table 2). Τhe most sophisticated and specific techniques used for the determination of isotope ratios are Isotope Ratio Mass Spectrometry (IRMS) and Site-Specific Natural Isotope Fractionation-Nuclear Magnetic Resonance (SNIF-NMR). Isotopic methods are based on the fact that natural products present measurable differences in specific proportions of particular isotopes, depending on their botanical origin and climatic and geographical conditions [32,33,34,35]. Additionally, stable isotope analysis can be used for the detection of added sugar and water in juice and for the differentiation between directly pressed juice and juice obtained from concentrates [34,36,37,38,39]. In particular, δ^13^C ratios can be indicative of the addition of sugars and sweeteners from C4 plants, like cane or corn, while added beet sugar can be detected through the determination of (D/H) ratios in the ethanol obtained from the fermentation of sugars [36]. Indication of the addition of tap water, along with geographical origin, can be provided by δ^18^O ratio and δ^15^N, which can also reveal the illegal addition of pulp wash. δ^15^N values mostly reflect the agricultural practices used and cannot be directly linked to geographical origin as they are influenced by soil nitrogen, coming mainly from fertilizers and manure [33]. 

The complementary implementation of IRMS and SNIF-NMR for the characterization of Slovenian and Cypriot fruit juices was presented in 2009 by Ogrinc et al. [34]. Values of δ^13^C in sugars, pulp and ethanol (obtained from the fermentation of the juice) and δ^18^O in water were determined by IRMS and (D/H)_I_ and (D/H)_II_ ratios by SNIF-NMR. The undeclared addition of cane sugar and beet sugar was revealed in 40% of the juice samples from Cyprus and in several Slovenian samples. The stable isotope data were statistically evaluated using PCA and clear discrimination was achieved between juices from the two countries, arising from their different precipitation regimens and climates. However, the identification of the botanical origin of fruit juices presented increased difficulty. In a more recent study of the same group, the discrimination between apple juices of different geographical regions of Slovenia and different apple cultivars (Idared, Golden Delicious and Topaz) was achieved through the isotopic and elemental characterization of 64 apple juice samples [35]. Combining both isotopes and elemental data together (12 variables: pulp δ^15^N and δ^13^C, water δ^2^H and δ^18^O, ethanol (D/H)_I_ and (D/H)_II_ and S, Cl, Fe, Cu, Zn and Sr), an overall 83.9% prediction ability of the geographical origin and 75.8% prediction ability of the cultivars was achieved. Alternatively, the applicability of sulphur- and strontium- isotope analysis in the discrimination of geographical origin of orange juice samples was evaluated by Rummel et al. pulp δ^2^H, δ^13^C, δ^15^N, δ^34^S and ^87^Sr/^86^Sr were measured in approximately 150 samples obtained from different orange cultivating regions around the world [33]. Discriminant Analysis (DA) was applied, using all pulp isotope ratios as variables, and 88.5% of the samples were correctly classified as samples from Mediterranean countries, South-America and Middle-America with Florida with δ^2^H and ^87^Sr/^86^Sr being the most discriminatory parameters. δ^34^S analysis results can be used to discriminate some sites of orange cultivation such as Cuba and Mexico. Moreover, Sr-isotope analysis proved to be a valuable tool for the detection of the undeclared addition of concentrate in single strength juices [33]. 

AIJN has set up threshold limits for various isotopes (δ^18^O_water_, (D/H)_I_, δ^13^C_sugars_, δ^13^C_ethanol_, δ^13^C_pulp_ and δ^13^C_acids_) in order to discriminate between authentic and non-authentic orange and lemon juices [8]. However, the actual values measured in genuine citrus juices may vary significantly depending on geographical origin – Bontempo et al. recommend the revision of these reference values, noting that AIJN thresholds are sometimes not fully applicable to samples of Italian origin, as indicated after performing a thorough isotope characterization of more than 500 samples of Italian citrus juices [38]. In the same study, samples of different geographical origin and harvest year could not be distinguished through the evaluation of these parameters, pointing out the need for further parameter determination (δ^34^S, ^87^Sr/^86^Sr, trace elements). Organic and conventional clementine juice samples were able to be distinguished using ^18^O/^16^O ratio [40]. 

Most recently, LC-IRMS technique has been used in authenticity assessment studies, demonstrating its great potential in this field [41,42]. LC-IRMS systems consist of a liquid chromatograph directly coupled to a liquid oxidation interface (based on organic molecules’ wet oxidation in aqueous solution and the production of CO_2)_ subsequently connected to an isotope ratio mass spectrometer with an electron ionization source [43]. The introduction of LC/IRMS has enabled the δ^13^C determination of specific compounds directly from complex mixtures, without any purification steps. In this context, the LC-IRMS determination of four Judgmental Ratios, linking the δ^13^C values of citric acid, tartaric acid, malic acid, glucose and fructose, was proposed by Bononi et al. to identify the undeclared addition of exogenous organic acids and sugars in lemon juice. Analysis of Italian market juice samples revealed a large-scale addition of exogenous materials [41]

#### 2.3.2. Elemental Analysis

The multi-element composition of juices is a powerful geographical indicator of the origin of the juice as it reflects the soil composition of the location fruits and vegetables have grown [35,44]. However, due to the large number of variables involved when the elemental profile is used as a chemical descriptor, advanced chemometric analysis is essential to evaluate the obtained information [45]. Gaiad et al. have realized a thorough research correlating geographical origin of Argentinean lemon juices to their elemental profile – 25 trace elements were determined using Inductively Coupled Plasma-Mass Spectrometry (ICP-MS) and five different multivariate analysis techniques were tested in order to classificate the samples according to their geographical origin [44]. Support Vector Machine (SVM) has been reported to have the highest classification rate of 76%, followed by Random Forest (RF) with 71%. Among trace elements determined in Slovenian apple juices, S, Cl, Fe, Cu, Zn and Sr have proved to be the most discriminatory parameters; nevertheless, both isotopes and elemental data must be combined together, using Linear Discriminant Analysis (LDA), in order to achieve efficient classification of apple juices according to geographical origin [35]. Organic and conventional grape juices can also be differentiated according their elemental profile, as shown by Borges et al. [46]. PCA and SIMCA models were developed, indicating that 11 elements predominated in the organic - conventional grape juice discrimination. Making one step forward, Maione et al. used different data mining methods, such as SVM, Neural Networks (NNs) and Decision Trees, to develop predictive models that could differentiate organic–conventional grape juice according to their multi-element composition. Na, K, P, Sn, Sm and Nd were the most significant variables and all the obtained models yielded an accuracy > 85% [47]. 

Apart from trace element patterns, individual values of specific elements like K, Na, Ca and Mg can be used as markers for the detection of the potential adulteration of fruit juices [25,30,37,48]. A low K concentration of orange juice is probably an indication of excessive dilution with water, as oranges are characterized by high K concentrations [25,37]. The same applies also for pomegranate juice: juices with K contents below 2000 mg L^−1^ are highly suspect of being adulterated, either by water addition or with other cheaper juices like grape or peach [49]. K/Mg ratios less than 50% can be an indicator of the addition of sweeteners in orange juice [48], while a high Na concentration can reveal the addition of preservatives or adulterants as salts [37]. Finally, high calcium levels can denote the adulteration of orange juice with pulp as pulp wash contains substantially more calcium than juice [37]. In every case, K, Na, Ca and Mg content alone is not enough to classify a juice as non-authentic and it needs to be combined with other atypical criteria [49]. 

### 2.4. Spectroscopic Techniques

Spectroscopic techniques based on UV-vis, fluorescence, infrared (IR), Raman and NMR spectroscopy are broadly used for food fingerprinting. During the last years, the applicability of spectroscopic techniques coupled with multivariate data analysis for the evaluation of food authenticity and the detection of adulteration has been widely demonstrated [15,32,50,51]. Non-targeted fingerprinting analysis uses fast spectroscopic techniques to obtain food fingerprints and presents a valuable alternative to chemical profiling methods. Advantages of these techniques comprise to little or no need for sample preparation, low cost, high reproducibility and non-destructive nature [16]. Recent applications of spectroscopic techniques in fruit juice authenticity assessment are presented in Table 3. 

#### 2.4.1. UV and Fluorescence Spectroscopy

UV spectroscopy is one of the most commonly used analytical techniques and it represents a very attractive choice for the assessment of food authenticity as it is simple, rapid and cost effective [71]. During the last decade, only two applications of UV-spectroscopy in authentication of fruit juices have been reported. In 2013, Boggia et al. proposed a rapid and easy-to-use screening method, based on UV–VIS spectroscopy and PCA, in order to single out potentially adulterated pomegranate juice samples (diluted samples or samples that contain undeclared cheaper juices like apple or grape) [52]. Satisfactory discrimination of pomegranate, apple, red and white grape juices was achieved and adulteration of pomegranate juice was detected down to 10% [52]. Most recently, Chang et al. have successfully applied UV spectroscopy and chemometrics for the determination of variety, adulteration, quality and ageing of apple juices [53]. PCA proved to be very useful to discriminate apple juice varieties and to detect adulteration. Principal Component Regression (PCR) and Partial Least-Squares Regression (PLSR) models were developed and their ability to predict the adulterant’s percentage, the diminution of quality due to storage and the time of storage of apple juices were compared [53]. 

Fluorescence spectroscopy is a very sensitive and selective technique, able to determine composition, nutritional and functional properties of food without using chemical reagents. Recently, the application of fluorescence spectroscopy in food authenticity studies combined with multidimensional statistical techniques is gaining increasing attention [72]. Two studies have been reported concerning the use of fluorescence spectroscopy in fruit juice authentication; the first uses 3D-Front-Face Fluorescence (3D-FFF) spectroscopy to determine orange juice adulteration with grapefruit juice [54]. With 3D-FFF Spectroscopy, the signal generated contains information about all the fluorescent compounds within the sample. According to Ammari et al., when 3D-FFF Spectroscopy is combined with Independent Components Analysis (ICA), the fraudulent addition of grapefruit juice to orange juice can be detected down to 1%, due to fluorophores detected in λ_ex_: 350–400 nm/λ_em_: 385–500 nm and λ_ex_: 300–380 nm/λ_em_: 360–460 nm that are much more intense in grapefruit than orange juices. [54] The second reported application describes the use of Synchronous Fluorescence Spectra (SFS) and Partial Least Squares Discriminant Analysis (PLS-DA) in the discrimination of apple juice obtained directly from fruit versus apple juice reconstituted from concentrate [55]. Synchronous Scanning Fluorescence technique holds many advantages in providing food fluorescent fingerprints comparing to conventional emission spectra or excitation-emission matrices. SFS are obtained by scanning simultaneously both the excitation and emission monochromators keeping a stable wavelength interval. Narrower and simpler spectra are obtained providing an improvement in selectivity for complex food samples [72]. Włodarska et al. used PLS-DA method to develop discrimination models for the two juice categories; very good predictive ability was obtained when pre-processed Total SFS and Specific SFS were used. PCA could discriminate the juices to some extent (14.8% of the total variation) but the most of the spectral variability seemed to be independent from the different juice categories [55]. 

As it can be clearly concluded, the potential of UV and fluorescent spectroscopy in fruit juice authentication has not been fully investigated. Both screening approaches have the advantages of being quick and relatively inexpensive to carry out and could prove to be very valuable in detecting suspicious samples that deserve further investigation, avoiding the extensive analysis of large numbers of samples.

#### 2.4.2. Vibrational Spectroscopy Techniques

Vibrational spectroscopic techniques are based on two fundamentally different phenomena, Infrared (IR) absorption and Raman scattering. Raman spectroscopy involves the inelastic scattering of light by a sample (gas, liquid, or solid). Upon irradiation, molecules change their vibrational state and this results in a corresponding change in the energy of scattered photons [73]. Contrarily, IR spectroscopy measures the absorbance of infrared radiation. Each functional group of a molecule has a unique vibrational frequency in the IR region and this can be used to define which functional groups are present in a sample. Thus, a unique molecular “fingerprint” is obtained for each sample and this fingerprint can be used to confirm its identity [50]. Even though both IR absorption and Raman scattering report on molecular vibrational characteristics, they provide complementary information since these two phenomena are controlled by different selection rules [73]. 

The IR region of the electromagnetic spectrum is divided into three zones: the near-, mid-, and far-infrared (NIR, MIR and FIR, respectively), categorized according to their relation to the visible spectrum. NIR spectroscopy (4000–10,000 nm) provides structural information about the vibrational behaviour of combinations of bonds and can be used for the elucidation of molecules. MIR spectroscopy (2500–25,000 nm) provides less complex structural information than NIR, mainly about the molecular bonds of a molecule, revealing which types of molecules are present in a sample [50]. MIR spectroscopy is commonly used as FTIR (Fourier Transform Infrared) spectroscopy, providing paramount chemical information about a food sample. FT-IR spectroscopy is most often used for the identification of unknown materials, based on the measurement of relevant fundamental vibrations [15].

During the last decade, the suitability of NIR and, particularly, FTIR in conjunction with multivariate analysis for the assessment of fruit juice authenticity has been repeatedly demonstrated. NIR spectrometry, combined with chemometric techniques (PCA, Principal component-radial basis function neural networks, PC-RBFNN) was used to discriminate pure bayberry juice with juice adulterated with water. After employing multiplicative scatter correction (MSC) and standard normal variate (SNV) transformation to pre-process NIR spectra, the results indicated that PC-RBFNN can distinguish authentic bayberry juice samples to water-adulterated samples (recognition rate: 97.6%) but cannot clearly estimate the levels of water in the adulterated bayberry juice [56]. The differentiation of grape juice varieties was reported by Cozzolino et al. using NIR and MIR spectroscopy combined with pattern recognition methods. PCA, LDA and PLS-DA were applied to grape juice samples classified according to variety (Australian Chardonnay and Riesling) based on both NIR and MIR spectra, using full cross-validation (leave-one-out) as a validation method. In total, LDA models classified correctly 86% and 80% of the grape juices, using MIR and NIR, respectively while the PLS-DA models produced an overall rate of 86% of correct classification [57]. 

He et al. used FTIR spectroscopy to develop a simple protocol for the classification of commercial apple, blueberry, cranberry, Concord grape and plum juices and their blends [58]. Sugar-rich and phenol-rich fractions of the juices were isolated following an SPE procedure and their IR spectra were used to construct the Soft Independent Modelling by Class Analogy (SIMCA) model for pattern recognition. The phenol-rich fraction model provided 100% correct classification of the juices and also zero percent of misclassification was achieved for cross-validation and external validation. However, the number of samples examined was rather limited [58]. The authentication of Concord grape juice, among other grape varieties (red, white and Niagara), in grape juice blends using FTIR was studied by Snyder et al. [59]. SIMCA provided satisfying discrimination among the different varieties of grape juices, mainly attributed to differences in IR bands between 1300 and 1800 cm^−1^, associated with specific vibrations of phenolic compounds. The Interclass Distance (ICD) values ranged from 17 to 41 for the different grape juice varieties, demonstrating the capacity of the varietal model (ICDs greater than 3 demonstrate good discrimination) [59]. Furthermore, the potential adulteration of pomegranate juice concentrate with grape juice concentrate can be detected using FTIR spectroscopy coupled with PCA [60]. The most important difference in the spectra of pomegranate and grape concentrate was observed in the 1700–1800 cm^−1^ region, corresponding to the C=O stretching mode. PCA was able to differentiate authentic pomegranate and grape juice concentrates and also to detect adulteration of the pomegranate concentrates down to 2%. In both cases, two principal components could explain 99% of the variability [60]. 

Besides juice-to-juice adulteration, FTIR spectrometry combined with chemometric analysis of spectral data was able to predict the adulteration of mango juice by sugar addition [61]. The detection limit for added sugar content varied from 3% (for samples with low natural total soluble solids, TSS) to 5% (for samples with natural TSS > 10%). The most accurate prediction model was obtained when using PLS regression in the range of 1476–912 cm^−1^ wavenumber, except for TSS which was best predicted using multiple linear regression (MLR) model on three specific wavenumbers (1088, 1050, 991 cm^−1^) [61]. Finally, two studies have been reported for the use of FTIR in the authentication of orange juices: Shen et al. studied the adulteration of freshly squeezed orange juices with 100% concentrated orange juices [63] while Ellis et al. reported the applicability of FTIR in the detection of water and sugars addition in authentic orange juice [62]. Adulterated orange juice samples were prepared with 0.5–20.0% water disguised with fructose, glucose or sucrose and FT-IR spectra in combination with PLSR were able to predict the levels of adulteration with very good accuracy (typical error: 1.7%) [62]. Freshly squeezed juice samples adulterated with different levels of concentrated juice were also correctly classified using LDA (overall prediction accuracy of 87.5%), however more samples including different varieties and origins should be incorporated in order to build a more robust prediction model [63].

#### 2.4.3. Nuclear Magnetic Resonance (NMR)

NMR spectroscopy is one of the most widely used analytical techniques for metabolomic studies and food fingerprinting [74]. It involves the analysis of the energy absorption of the atomic nuclei with non-zero spins in a magnetic field’s presence. The atomic nuclei’s energy absorptions are influenced by the nuclei of surrounding molecules, causing small modifications to the external magnetic field [32]. NMR spectroscopy is a very powerful technique for structure determination as it can provide exhaustive information concerning the molecular structure of a food sample along with high-throughput spectroscopic/structural information of the metabolites present in the sample [75]. Its main advantages encompass its non-invasive nature, high accuracy and precision, ease at use and rapid acquisition of data. On the other hand, NMR spectroscopy presents low sensitivity compared to other analytical techniques (FT-IR or MS) and the instrumentation is very expensive with high running costs [74]. Most NMR studies are based on ^1^H measurements over ^13^C, most likely because of the higher sensitivity and shorter relaxation times. 

High-resolution NMR, with frequencies more than 100 MHz, has been more widely applied in food authentication than low-resolution NMR (frequencies of 10–40 MHz), as it can provide more detailed information regarding the molecular structure of food metabolites [71]. The NMR spectra of food have a high level of complexity, containing numerous signals related to primary and secondary metabolites. Hence, multivariate analysis is necessary, in most cases, to extract the required information [12]. Sobolev et al. have reviewed in 2015 the use of NMR methodology for fruit and fruit juice characterization and have reported several metabolites that have been identified using untargeted NMR approaches [75]. Moreover, several additional studies using NMR spectroscopy have been conducted in the last years regarding fruit juice authenticity [64,65,66,67,68,69,70]. 

A screening fruit juice profiling method using ^1^H-NMR was developed by Bruker BioSpin GmbH and SGF International e.V. and has been introduced since 2009 under the name Spin Generated Fingerprint (SGF)-profiling [64]. This method was suggested as a method for fruit juice authentication and quality verification, including both target and non-targeted analysis of more than 6,000 reference juices (1,500 fully authentic samples). This approach allowed the quantification of more than 28 compounds, depending on the type of juice, along with an automated non-target screening using statistical models for the assessment of fruit content, type and origin of juices. Furthermore, other frauds like the undeclared addition of sugar or the extraction of orange peel in orange juice, were able to be revealed through the determination of specific markers (like sucrose, galacturonic acid, phlorin) [64]. Another study regarding the detection of juice-to-juice adulteration was reported by Vigneau and Thomas [65]. The classification of authentic orange juices and orange juices adulterated with clementine juice, using the ^1^H-NMR spectroscopic profiling and PLSR, was evaluated. Various preprocessing strategies and variable selection procedures were tested and the lowest error rate was obtained using logarithm and Pareto scaling, based on backward interval PLS or on genetic algorithm [65]. 

NMR spectroscopy has also been used for the differentiation of fruit juice varieties [66,67,68] and the investigation of storage conditions in juice quality [68,69]. Different clones and cultivars of sour cherry juices were differentiated using ^1^H-NMR spectra and a PLS-DA model with prediction ability more than 82%. Additionally, the correlation between NMR data and sensory analysis indicated that the content of malic acid had a significant influence on the categorization of juices as sour or sweet. The content of glucose only slightly affected and of fructose did not affect at all these attributes [66]. Nevertheless, this study presents limitations, mainly due to the low number of samples (seven overall, one from each cultivar), which weakens the validity of the models. The discrimination of mango juices belonging in five different cultivars was realized using band-selective excitation ^1^H-NMR spectra and PCA [67], while orange juice variety discrimination was achieved by De Oliveira et al., using specific metabolites and PCA, in five different orange varieties: Pêra Rio, Bahia, Murcote, Lima and Persian Lima [68]. 

The main objective of De Oliveira’s study, however, was the quality control of orange juices through the NMR determination and evaluation of the major chemical constituents under different storage time and temperature conditions. It was found that storage up to 24h led to the production of formic, fumaric, succinic, acetic and lactic acid and also to the appearance of ethanol which was not detected at 0 h. Moreover it was found that the production of these compounds is increased when juices are stored at 24 °C than at lower temperature (14 °C), as expected [68]. The production of ethanol was also found to be significantly increased in grape juices stored for 6 days at room temperature or 12 days in the refrigerator, indicating that fermentation process caused by microorganisms is enhanced at warmer temperatures [69]. The highest increase in ethanol content was detected in sweetened juices with no preservatives, showing that the addition of sucrose enhances fermentation but also pointing out the necessity of preservatives’ addition, at least at sweetened juices. Overall, the analysis of grape juice samples with NMR, without sample pre-treatment, was able to provide significant information concerning storage time and temperature but also to detect undeclared addition of citric acid, sucrose and preservatives [69]. 

Finally, untargeted ^1^H-NMR fingerprinting was used by Longobardi et al. for the geographical origin characterization of Italian sweet cherry juices [70]. Initially, PCA was applied to differentiate samples from two different regions of Italy (Emilia Romagna and Puglia). However, only 21% of data could be explained by the first three principal components and so PCA was considered unable to distinguish samples from different origins. On the contrary, PLS-DA and LDA were able to successfully discriminate cherry juices of the 2 origins with external validation procedure showing prediction abilities equal to 94.9% for PLS-DA, and to 92.3–94.9% for LDA models [70].

### 2.5. Separation Techniques

Separation techniques, and particularly liquid chromatography (LC), gas chromatography (GC), capillary electrophoresis (CE) and thin layer chromatography (TLC), are widely used in food authenticity studies as they can provide rapid and reliable separation of molecules with very similar chemical characteristics, even in an extremely complex matrix as food. They can be connected to various detectors to detect and identify analytes, with mass spectrometric detection being the most extensively used in foodomics due to its excellent compound identification capabilities. 

#### 2.5.1. Thin Layer Chromatography

Thin layer chromatography is an easy-to-operate, rapid and cost-effective chromatographic technique which has been widely used in food analysis for several decades [76,77]. Its main advantages compared to column chromatography comprise to its simplicity, minimum demands for sample preparation and solvent consumption and the possibility to simultaneously analyze multiple samples side-by-side. TLC, coupled with UV, densitometric and mass spectrometric detection, has been used to determine several classes of fruit juice constituents such as carotenoids, vitamins, sugars and polyphenols [76,77]. However, so far, there are only few applications of TLC to fruit juice authenticity assessment and quality control (Table 4). Filip et al. used TLC-densitometry to establish berry juice authentication based on anthocyanin and anthocyanidin fingerprints. The method was successfully applied to juice samples obtained from Romanian markets aiming to verify their conformity with the declared label [78]. TLC on silica gel 60 F plates was used to detect 3’5’-di-C-β-glucopyranosylphloretin (PD), a marker declaring shiikuwasha juice adulteration with calamondin juice [79] and also for the separation of 11 anthocyanins in grape juice, followed by identification with mass spectrometry. Obtained anthocyane patterns proved to be highly comparable for juices from the same plant source while markedly different between plant sources (for example black- currant juice was clearly different from elderberry juice) [80].

#### 2.5.2. Capillary Electrophoresis 

CE is a technique that separates molecules according to their electrophoretic mobilities, depending on a molecule’s charge and size. The velocity with which a charged molecule can travel down a capillary is contingent on its electrophoretic mobility and the applied electric field. Higher voltages lead to faster velocities and, therefore, faster separations. CE has various separation modes, such as capillary zone electrophoresis (CZE), capillary gel electrophoresis (CGE), capillary isoelectric focusing (CIEF), electrokinetic chromatography (EKC), micellar electrokinetic chromatography (MEKC) and non-aqueous capillary electrophoresis (NACE). CE enables the separation of a wide range of compounds ranging from small molecules, such as amino acids or biogenic amines, to large biopolymers, such as proteins and DNA [81]. CE hasn’t been widely used for food authentication so far, even though it is a powerful separation technique for charged metabolites (Table 4). Its main drawback is that it is not as robust as GC or LC and the retention time of the analytes can shift, even among the repeated analysis of the same samples. This could generate significant problems associated with data processing (in peak picking or peak alignment), undercutting metabolomic studies [82]. 

Herrero et al., Castro-Puyana et al. and Vallejo-Cordoba et al. have reviewed the most important CE applications in food science and technology, including food safety and quality control, nutritional value, food processing and storage impact [83,84,85]. These reviews describe several applications of CE in fruit juice’s analysis reported till 2011, such as the determination of aminoacids, organic acids, catechins, L-carnitine, patulin, fungicides, fructose/glucose and citric acid/D-isocitric acid ratio determination, which are important fruit juice adulteration markers. Most recently, Tezcan et al. used a new chiral micellar electrokinetic chromatography-laser induced fluorescence (MEKC-LIF) method for the determination of chiral aminoacids in pomegranate juices. L-Asparagine was proposed as a marker indicating the adulteration of pomegranate juices with apple juices [86]. Free aminoacids were also determined in passion fruit juices using CE-UV and the quantification of six of them (proline, glutamate, isoleucine, leucine, phenylalanine, arginine), combined with PCA, was able to characterize passion fruit juices according to their industrial provenance (natural juice, concentrated juice, box juice, organic juice, frozen pulp and mixed fruits juice) [87]. CZE with indirect UV detection was also used for the determination of sugars profile and aliphatic organic acids in fruit juices and nectars. Sugar concentration ratios and organic acid concentration ratios were used as predictors to construct LDA models for the classification of different fruit juices and all juices were correctly assigned within a 95% probability level [9,88]. Multiple Linear Regression (MLR) was used to detect and quantify juice blends according to sugar ratios obtained; average prediction errors were < 4.0%, indicating the applicability of the constructed MLR models [88]. 

#### 2.5.3. Chromatographic Techniques–Mass Spectrometry

Chromatography is the most widely used separation technique in food analysis. Its main advantage is that it provides both the separation of the constituents of a complex sample and also, when combined with a powerful detection technique such as Mass Spectrometry (MS), the identification of these molecules. Gas chromatography (GC) is suitable for the determination of volatile and semi-volatile compounds, while liquid chromatography (LC) is a more versatile technique, allowing the determination of a wider variety of compounds [10]. 

During the last decade, MS has emerged as the foremost technology in food authenticity studies due to its unique sensitivity and specificity. The paramount complexity and the wide concentration range of food samples present substantial challenges for any novel analytical methodology and, in this context, the use of high and ultra-high-resolution mass spectrometers has led to a great improvement of the analytical performance [89]. MS-based authenticity studies are performed using two distinctive workflows: target analysis and non-target analysis. Targeted approaches focus on the determination of one or a small number of compounds, that can be used as authenticity markers, and for which their possible identity is already known before analysis. On the contrary, untargeted approaches refer to the determination of the total set of food metabolites, without bias, in order to be used for the evaluation of food authenticity, without the need for individual markers’ identification [89]. 

Numerous fruit juice authenticity studies have been reported using both GC and LC, coupled with MS detection and also with different types of detectors such as Flame Ionization Detector (FID), Diode Array Detector (DAD), Refractive Index Detector (RI), and Fluorescence Detector (FLD). 

##### GC Methodologies

In most fruit juice authenticity studies the volatile compound profile of fruit juices is determined using GC-MS methodologies, while GC methods using conventional (non-MS) detectors are very scarce. Both targeted and untargeted GC-MS approaches have been followed in order to discriminate the geographical origin, the varietal origin and the potential adulteration of apple, orange, pomegranate, pear, lemon and shiikuwasha juices (Table 4). 

Targeted GC methodologies, using mainly headspace-Solid Phase Microextraction (HS-SPME) as extraction method, have been used for the identification and quantification of specific groups of juice volatile compounds such as alcohols, esters, terpenes, aldehydes, terpenoids, hydrocarbons, acids, sulfur compounds and ketones [37,49,79,90]. Various compounds belonging in these classes have been identified as markers of juice-to-juice adulteration; for example, the presence of linalool and linalool oxide at concentrations exceeding 3% and 0.10% in pomegranate juices indicates the addition of grape juice, while elevated concentrations of isoamyl butyrate, isobutyl butyrate, benzyl acetate and butyl acetate are indicative of the addition of peach juice [49]. Linalool and γ-terpinene, have also been identified as markers for the detection of shiikuwasha juice mixed with calamondin juice [79,91]. As reported by Willems and Low, the oligosaccharide profile of pure and adulterated pear juices can be used to detect the addition of High Fructose Corn Syrup, Total Invert Sugar and Hydrolyzed Inulin Syrup in authentic pear juices at levels of 0.5−3.0% (*v*/*v*) [90]. The capillary gas chromatography-flame ionization detection (CGC-FID) method used for oligosaccharides’ determination can also be used for the determination of arbutin, exploiting the juice-to-juice debasing of apple juice with pear juice. GC-FID was also used for the untargeted profile analysis of volatile organic compounds (VOCs) in lemon juice, in order to investigate the adulteration of organic with industrial lemon juice. A linear relation between the percent of organic lemon juice and chromatogram characteristics like total peak area, peak height and peak number was observed, suggesting the applicability of this methodology in the detection of organic lemon juice adulteration [92]. 

Apple juice authentication studies using untargeted GC-MS approaches have been also reported during the last years, mainly focusing in the varietal and geographical origin discrimination of the juices [93,94]. In a novel GC-MS untargeted approach, retention times variables were obtained using the chromatographic fingerprints of apple juice samples of six different varieties [93]. A Stepwise Linear Discriminant Analysis (SLDA) classification model was developed and achieved to discriminate apple juice varieties with 100% recognition ability and prediction ability. Ten retention times were selected by SLDA to significantly attribute to the variety discrimination and these ten volatile compounds were identified using GC-MS spectra. Similar approach was used for the classification of apple juices according to the geographical origin: SLDA was used to differentiate samples from 4 China counties and a recognition ability of 93.9% and a prediction ability of 89.8% were attained. Butyl acetate, (*E*)-2-hexenal, hexyl 2-methylbutanoate and pentyl acetate were identified as informative markers to contribute to geographical origin discrimination of apple juices [93]. Most recently, the classification of eight different apple juice varieties (Fuji, Golden Delicious, Qin Guan Delicious, Pink Lady, Starkrimson, Jonagold, Gala and Ralls Janet) based on their volatile components was attempted by Wu et al. and forty-four volatile components were tentatively identified [94]. Each variety could be discriminated from the others using marker compounds more or less specific to the varieties; for example, (S)-(-)-2- methyl-1-butanol and 1-dodecanol were detected only in Starkrimson apples, linalool oxide in Pink Lady, ethyl 2-methylbutyrate and ethanal in Qin Guan Delicious, methyl butyrate in Ralls Janet and methyl acetate in Jonagold [94]. 

Orange juice is the most popular fruit juice, representing 50% of worldwide consumption, and its authentication and quality assurance represents a very important issue for consumers. Different methodologies have been reported using GC-MS to evaluate orange juice quality, some of them connecting the volatile profile of orange juices with flavor and aroma [37,95,96]. In this context, a new flavor index was proposed by Schmutzer et al. calculated as the total sesquiterpenes of a juice sample divided to total terpenes (without limonene). A threshold value of a minimum of 40% is proposed to categorize orange juices from concentrate as high or lower value [37]. The presence of benzoic acid and D-limonene in orange juice reveals food flavors addition [95] while total alcohols and total ketones can be used to differentiate freshly squeezed orange juices with commercial orange juices and orange nectars, as the high concentrations of alcohols and ketones is indicative of sample processing and thermal treatment [37]. Varietal discrimination of orange juices was also achieved using their volatile profile in combination with new approaches based on Artificial Neural Networks (ANN) and Genetic Algorithms (GA). When GA was combined with LDA and with the Kohonen map, individual markers were identified (geranyl acetate for LDA and a-pinene and sabinene for the Kohonen network) that were able to discriminate the juices with a perfect match [96]. 

##### LC Methodologies

With the application domain of LC being much wider than the one of GC, High Pressure Liquid Chromatography (HPLC) and Ultra High Pressure Liquid Chromatography (UHPLC) has been extensively used in fruit juice authenticity studies (Table 5 and Table 6). (U)HPLC has been applied to the separation and quantification of fruit juice chemical constituents, mostly phenolic compounds, organic acids, sugars and amino acids, for characterization, classification and authenticity assessment purposes. The constantly increasing use of LC hyphenated with High Resolution MS (HRMS) analyzers, has led to an immense evolution of LC MS-based authenticity approaches presenting ideal performance characteristics for high-throughput food authenticity applications [89]. 

Targeted Approaches

Targeted LC methodologies for the determination of phenolic compounds, organic acids, aminoacids and sugars present a useful tool for authenticity and quality control of fruit based products. Through the determination of specific compounds belonging in these classes, several unique markers have been determined for individual fruit juices and have been used to assess fruit juice authenticity (Table 5).

One method to detect the fraudulent addition of a fruit juice to another, higher value, fruit product is through the determination of its phenolic profiling. Fruit juices’ phenolic profiles present consistent differences and can be used to identify which fruit is present in a product. In this context, a polyphenol characterization study of red fruit and vegetable juices (strawberry, blueberry, red grape, red raspberry, blackcurrant, European cranberry, sour cherry, purple prickly pear and purple carrot juice), using HPLC with simultaneous UV–Vis and fluorescence detection, was presented by Obon et al. [97]. Characteristic anthocyanin and betacyanin profiles were revealed for each juice and they could be considered as a ‘‘fingerprinting’’ of the specific juice. 

Citrus polyphenolic profiles have been also thoroughly studied with the aim of differentiating orange, mandarin, lemon and grapefruit juices. Indeed, Abad Garcia et al. have presented a comprehensive study using HPLC-DAD-ESI-MS/MS to determine 49 polyphenols in citrus juices from 18 Spanish cultivars. Naringenin-O-rhamnosylmalonylhexoside, naringenin-*O*-hexosylhexoside, naringenin-7-*O*-neohesperidoside-4-*O*-glucose, naringenin-7-*O*-neohesperidoside, hesperetin-7-*O*-neohesperidoside, hesperetin-7-*O*-rutinoside, iso-sakuranetin-7-*O*-neohesperidoside, apigenin-7-*O*-neohesperidoside, apigenin-6-*C*-hexoside-*O*-hexoside and scopoletin-*O*-hexoside were found to be characteristic of grapefruit juice while eriodictyol-7-*O*-rutinoside, eriodictyol-7-*O*-rutinoside-4-*O*-glucoside, diosmetin-8-*C*-glucoside, diosmetin-6-*C*-glucoside, diosmetin-6,8-di-*C*-glucoside, diosmetin-6,8-di-*C*-hexosideacylhexoside and luteolin-7-*O*-rutinoside have been named as lemon juice markers [98]. It is well known that the most common fraudulent practice for citrus juices adulteration is the addition of lemon juice, grapefruit juice or mandarin juice to orange juice and so these markers can present a reliable tool to detect this type of adulteration [98]. Neo-hesperidin and naringin could be used as unique markers to trace adulteration of orange juice (Jaffa and Mosambi varieties) with Red Blush grapefruit juice at a lowest adulteration level of 2% [6] and naringin, neoeriocitrin, and neohesperidin can also be considered as indicative of the fraudulent addition of bergamot juice to authentic lemon juice [26]. 

Juice-to-juice adulteration of pomegranate juice can also be detected by interpreting its anthocyanin profile: Turkish pomegranate juices were found to contain six specific anthocyanins: delphinidin-3,5-diglucoside, cyanidin-3,5-diglucoside, delphinidin-3-glucoside, pelargonidin-3,5-diglucoside, cyanidin-3-glucoside and pelargonidin-3-glucoside. This anthocyanin profile of pomegranate juices, determined with HPLC-DAD, was very consistent and almost irrelevant of the fruit variety or geographic origin [29]. In this context, several grape, sour cherry and red-skin apple anthocyanins could be used for the identification of pomegranate juice adulteration: the determination of malvidin-3-glucoside, malvidin-3,5-diglucoside, peonidin-3,5-diglucoside and peonidin-3-diglucoside reveals the adulteration with grape juice, the determination of cyanidin-3-sophoroside, cyanidin-3-glucosylrutinoside, cyanidin-3-rutinoside and cyanidin-3-glucoside is indicative of sour cherry addition and the determination of cyanidin-3-galactoside, cyanidin-3-arabinoside, cyanidin-7-arabinoside, cyanidin-3-rutinoside, cyanidin-3-xyloside and cyanidin-3-glucose declares the adulteration of pomegranate juice with apple juice [29]. A similar study of pomegranate anthocyanins by Borges et al. also resulted in a straightforward methodology of pomegranate juice authenticity evaluation through the HPLC–DAD–MS detection of red grape constituents [99]. Additionally, the debasing of both pomegranate and red grape juice with apple juice can be reliably detected through the determination of phloridzin, a flavonoid that has been identified as a typical apple juice marker [100]. 4-*O*-p-coumarylquinic acid has been reported as an apple juice unique compound that can reveal the addition of apple juice to pear juice while isorhamnetin-3-*O*-rutinoside, abscisic acid and arbutin were identified as characteristic pear compounds [101]. 

The concentration levels of these compounds were found to vary significantly among juices from China and juices from other countries, allowing also the geographical origin verification of pear juices [101]. Geographical origin and varietal discrimination of apple juices has also been achieved through the phenolic composition determination combined with statistical data evaluation techniques. In a most recent study, Bizjak Bat et al. determined both primary and secondary metabolites of apple juices in order to investigate the possibility for regional and varietal discrimination of three apple cultivars from five different geographical regions in Slovenia. Several flavanols and flavonols were identified as reliable markers for the geographical origin differentiation of juices while an LDA model, including all determined compounds, managed to distinctively separate the three apple cultivars examined [102]. Similar results had also been reported from Guo et al. in 2013, who accomplished a satisfactory classification of apple juices, according to both cultivar and geographical origin, using SLDA and giving a 98.3 and 91.2% prediction ability success rate, respectively [103]. According to Guo et al., major flavan-3-ols, especially epicatechin, catechin and procyanidin B1 affect significantly the geographical origin differentiation while flavonols, including quercetin-3-*O*-glucoside, quercetin-3-*O*-rhamnoside and quercetin-3-*O*-arabinoside, were predominant in variety-based classification. 4-*p*-coumaroylquinic acid and 4-caffeoylquinic acid were found to influence significantly both geographical origin and variety discriminations [103].

Finally, several individual phenolic compounds have been confirmed to reveal the freshness and ageing of fruit juices like the neolignans isoamericanol A and isoamericanoic acid A which can be used to distinguish fermented and freshly squeezed juices [104] and 5,6- and 5,8-epoxycarotenoids which can be used to estimate juices’ freshness [105]. Overall, authenticity studies based on the evaluation of the phenolic profile of juices are pretty promising, and such analytical methodologies could be doubtlessly applied in fruit juice quality control. However, it must be taken into consideration that the climatic conditions, the environment, the processing procedure and even the degree of fruit ripeness can play a decisive role in the overall polyphenolic profile of juices and so the actual applicability of phenolic-based authenticity studies needs constant research [105]. 

Fruit juice aminoacids, organic acids and sugars have also been used as markers of juice authenticity and quality. Different types of fruit juice adulteration can be revealed by estimating the total amino acid value; for example, a decreased concentration of total amino acids can be indicative of either dilution with water or addition of sugar syrup [106]. The measurement of specific aminoacids and the characterization of amino-acid profiles of individual juices can be used to estimate juice quality, juice-to-juice adulteration and also the undeclared addition of in-expensive amino acids, such as glutamic acid or glycine, which are added in order to increase the total amino acid content [106].

D’ Orazio et al. refer to the determination of d-amino acids in orange juices as suggestive of low-quality juices, since high quality orange juices contain exclusively l-amino acids [107], and, most recently, Nuncio-Jáuregui et al. have related grape juice addition to pomegranate juice with an important increase in proline’s concentration levels [49]. 

In the same study, sugar profiles were used to detect pomegranate juice adulteration with peach juice; in pomegranate juice the predominant sugar was found to be fructose, with sucrose present only at trace levels, while peach juice presented a completely different profile with sucrose prevailing. Correspondingly, the adulteration of pomegranate with peach juice was able to be detected up to 10% (*v*/*v*), as sucrose content increased significantly. The combination of sugars profile, organic acids and specific physicochemical parameters data (pH, titratable acidity, total dissolved solids (TDS) and electrical conductivity (EC)) with Multivariate Analysis of Variance (MANOVA) and LDA resulted in a successful classification of Merlin Oranges from four different Greek regions with a correct classification rate of 83.3% [109]. Especially organic acids present advantageous authenticity markers in fruit products, as they are less susceptible to alterations during juice process and/or storage, in comparison to other juice constituents. In this context, grape juice presents elevated concentrations of tartaric acid while apple juice is characterized by its high quinic acid content. Based on their organic acids profile, the addition of both juices in pomegranate juice can be detected at levels down to 5% [49,108,110]

Untargeted Approaches 

The development of novel untargeted analytical approaches, complementary to targeted analyses, is completely essential in order to facilitate a more comprehensive insight into fruit juices’ chemical composition. Untargeted approaches can lead to more efficient methodologies for quality control and detection of adulteration since targeted methodologies can only be successfully applied to monitor a limited number of specific adulteration practices [111]. Modern HRMS techniques (QTOF/MS, Orbitrap), combined with UHPLC, are used to generate large datasets on thousands of analytes for which no previous knowledge exists. In order to process and evaluate these complex datasets, advanced data mining and data processing algorithms are needed, along with multivariate chemometric tools such as PCA, LDA, OPLS-DA and SVM [7]. The combination of HRMS-untargeted analyses and chemometrics provides the possibility of constructing discrimination models, on an untargeted basis, with subsequent identification of the specific marker compounds that have been found to significantly contribute to this discrimination [112] (Table 6).

The first untargeted methodology for pomegranate juice adulteration detection was reported by Borges et al. in 2010 [113]. Borges used an HPLC-DAD-MS/MS methodology to compile a ‘‘pomegranate juice polyphenolic fingerprint’’ and then compare it to those obtained from blended drinks. 17 compounds were tentatively identified, using MS and MS/MS spectra, in all 100% pomegranate juices and they were semi-quantified as cyanidin-3-*O*-glucoside equivalents. In a subsequent untargeted HPLC-DAD-QTOF/MS study, chlorogenic acid and one of its isomers were identified by Twohing et al. as markers of pomegranate juice adulteration, coming in agreement with other previous publications [101]. 

Citrus juices represent a food commodity often targeted for adulteration. In most cases, orange juice, being the most expensive citrus juice, is blended with cheaper juices like grapefruit or lemon. The detection of citrus juices’ adulteration is particularly challenging due to the similarity of citrus juices in terms of organoleptic and physicochemical characteristics and molecular composition. In this context, several untargeted authenticity studies have been reported in the last decade, aiming in the identification of new citrus juice authenticity markers and the development of novel prediction models to evaluate authenticity and quality. The majority of these methodologies focus on the discrimination of citrus juices according to their HR-MS metabolic profile and the detection of juice-to-juice adulteration [6,7,111,114]; nevertheless, the differentiation of varieties, origin and organic type of production has also been presented [112,114]. 

In a very comprehensive demonstration of the potential of untargeted screening approaches, Jandric et al. have published three considerable studies presenting the successful discrimination of different fruit juices and also orange juice varietal and geographical origin classification [6,7,112]. The metabolic profile of juices was obtained using UHPLC-QTOF/MS, in both positive and negative ionization mode, and mass spectrometric data were processed using unsupervised and supervised pattern recognition techniques (PCA, OPLS-DA and PLS-DA). PCA was able to distinguish between pineapple, orange, grapefruit, apple, mandarin, and pomelo juices [6,7] and also between oranges of different varieties (navel, lane late, valencia and navelina) and geographical origins (Spain, Greece and Italy) [112]. Discrimination was found to be better explained in negative ionization mode, presenting stronger model prediction values and juice-to-juice adulteration was able to be detected at very low levels, down to 1%. In all three studies, OPLS-DA and PLS-DA supervised techniques were subsequently used to evaluate the method’s feasibility and to elucidate the most influential markers that contribute most significantly to juices classification. Reliable and robust classification models were developed with recognition ability up to 100%, and several individual biomarkers were identified and confirmed using standard reference materials [6,7,112]. 

Further untargeted metabolomic studies evaluating fruit juice authenticity have been reported by Vaclavik et al., Arbona et al., Cuevas et al. and Wang et al. [111,114,115,116]. Vaclavik et al. used low resolution HPLC – QqQ/LIT MS data, combined with PCA, to group orange, apple and grapefruit juice samples. Twenty four Principal Components (PCs) were overall calculated, managing to explain 90% of total variance [111]. PCA loadings plots revealed in total 20 peaks that contributed significantly in the clustering of samples of specific fruit juice types. Subsequently, the majority of these compounds were tentatively identified using HR-MS (HPLC–QqTOFMS) data and mass spectral libraries matching. In addition, a supervised LDA model was developed to explore the potential of detecting the addition of apple and grapefruit juice in orange juice. The model succeeded in indicating the adulteration of orange juice at a percentage of 15% [111]. 

The secondary metabolite composition of citrus fruits was studied by Arbona et al. using untargeted LC/ESI-QTOF-MS metabolite profiling [114]. Secondary metabolites can be used either as markers for the characterization and discrimination of specific fruit commodities or as quality indices. Multivariate analysis (Hierarchical Cluster Analysis (HCA) and PLS-DA) was used to attain separation of different citrus juices such as orange juices, lemon, mandarin and grapefruit juices and also the varietal differentiation within a particular fruit type (blonde and navel oranges). Flow charts depicting biosynthetic pathways were also presented [114]. Metabolomic fingerprinting, volatile compound profiles and advanced chemometrics were combined from Cuevas et al. in order to authenticate organic premium orange juices; to the best of our knowledge this has been the only untargeted mass spectrometric study concerning the certification of organic juice production [115]. PCA and HCA unsupervised techniques did not provide distinctive discrimination of organic and conventional orange juices but when different data fusion approaches and PLS-DA were used the discriminant power of the model reached 100% [115]. 

## 3. Conclusions and Outlook

From the reviewed studies, it can be clearly concluded that considerable research has been performed since 2008 in the field of fruit juice authentication and quality control. Substantial progress has been made using state-of-the-art technologies and advanced analytical methodologies have been developed that can provide a powerful tool for the determination of fruit juice adulteration and the confirmation of varietal and geographical origin. Target and untargeted approaches have been developed using a series of analytical techniques such as chromatography, spectroscopy and DNA-based techniques. The majority of recent applications involve the use of newest technologies, such as HRMS, ICP-MS, SNIF-NMR and sophisticated multivariate statistical techniques, such as PCA and PLS-DA, for pattern recognition and development of classification and prediction models. 

Despite this vigorous on-going research, there is still room for progress on both analytical developments and data processing techniques. Ion mobility Spectrometry (IMS) is a novel separation technology that has been successfully interfaced with MS and can effectively separate compounds with similar structures, including chiral mixtures and isomers. It can be used for the identification of authenticity biomarkers in food and the establishment of MS/MS fragmentation pathways of isomeric natural compounds [117]. Multi-dimensional techniques, such as LC × LC or GC × GC are expected to be more widely applied in food authenticity and metabolomics studies, as they increase the number of peaks and also enhance resolution, selectivity and sensitivity compared to conventional chromatographic techniques [12]. As far as chromatographic separation is concerned, future prospects include the use of Hydrophilic Interaction Liquid Chromatography (HILIC) as a complementary tool for the determination of polar authenticity markers of fruit juices. So far, only one application of HILIC in fruit juice authenticity assessment has been reported, regarding a fast screening method for the determination of preservatives, artificial sweeteners, colorants, sugars and polyphenolic compounds in various fruit juices [118]. HILIC’s potential to provide novel chromatographic fingerprints of polar fruit juice compounds has not been yet exploited. 

Moving one step forward, the development and application of rapid, non-destructive analytical methods for food authentication with the minimum processing time and cost per sample is of prime importance. Direct Sample Analysis (DSA) combined with mass spectrometry can rapidly provide the characteristic mass spectral fingerprint of foods and could be a valuable tool in monitoring food fraud. Nevertheless, the implementation of DSA combined with mass spectrometry in fruit juice authenticity studies has been extremely scarce; to the best of our knowledge, there are so far only two studies regarding pomegranate and apple juice authentication using DSA-TOF/MS and APCI-MS, respectively [119,120]. Further research is most certainly expected to be realized in this direction, combined with more thorough studies concerning the different levels of data fusion and data processing techniques. Data fusion is tightly related to the performance of models generated, increasing the prediction/classification ability and decreasing the uncertainty of results [10]. So far, low-, and mid-level fusion approaches are most commonly used but novel hybrid approaches that combine both low- and mid-level data fusion, are emerging. Additionally, besides standard one - dimensional data multivariate approaches (PCA), specialized multi-block methods like Consensus PCA and Common Component and Specific Weight Analysis (CCSWA) will be extensively used in the near future to perform high-level data fusion [121]. 

To conclude, the literature reviewed in this paper indicates that various analytical methodologies have been developed to monitor and evaluate the major fruit juice authenticity issues like the fraudulent addition of water and sugars, juice-to juice adulteration, false labelling or the use of juice concentrates. However, there are no reported applications that implement these methodologies in official fruit juice quality control or routine analysis [15]. It is of paramount importance that novel, food fingerprinting methodologies came to be adopted by both industry and inspection agencies to monitor the fruit juice products and evaluate their authenticity. The challenges in the potential application of food fingerprinting approaches in the official food control involve the establishment of harmonized operating procedures, the standardization of both chemical analysis and statistical evaluation and the development of appropriate validation protocols of the statistical models [74]. 

## Figures and Tables

**Table 1:** Applications of DNA-based techniques in authenticity studies of fruit juices.

Fruit Juice	Aim of Study	DNA-based Method	Level of Detected Adulteration	Markers	Ref.
Orange, Mango, Peach, Pear, Pineapple	Fruit species discrimination in fruit juices	HRM analysis	25%	DNA barcode trnL	[18]
Pomegranate	Detection of adulteration with bulking agents	SCAR markers	1%	ScAc_336_, ScAm_358_, ScDa_185_, ScEo_143_, ScMd_262_, ScMn_293_, ScSn_226_, ScVma_241_, ScVmy_287_ and ScVv_144_	[19]
Orange	Detection of adulteration with mandarin juice	Dual-probe real time PCR	5%	Single-nucleotide polymorphism at the chloroplast chromosome trnL–trnF intergenic region	[20]
Orange	Detection of adulteration with mandarin juice	Dual-probe real time PCR	1%	Mandarin-specific polymorphismin chloroplast trnL gene	[21]
Orange	Detection of adulteration with grapefruit and mandarin juice	PCR - Laboratory-on-a-chip Capillary Electrophoresis (LOC).	10% and 2.5%, respectively	PCR restriction fragment length polymorphism (RFLP) assay (grapefruit) and PCR heteroduplex assay (mandarin)	[22]
Grape, Apple, Pear, Peach, Strawberry, Orange, Mandarin	Discrimination of fruit juices	PCR, real-time PCR and DHPLC	10%	ITS1-5.8S-ITS2, TrnL-TrnF from chloroplast genome and thaumatin-like protein gene from nucleus genome	[23]

DHPLC: Denaturing High Performance Liquid Chromatography, HRM: High Resolution Melting, PCR: Polymerase Chain Reaction, SCARs: Sequence-Characterized Amplified Regions.

**Table 2:** Applications of isotope and elemental techniques in authenticity studies of fruit juices.

Aim of Study	Analytical Technique	Type of Study	Chemometric Approach	Sensitivity & Accuracy	Authenticity Markers	Ref.
Authentication of orange-based fruit juices - Identification of exogenous addition of water & sugar	ICP	Targeted	-	-	K, Ca, Brix value	[25]
Authentication of pomegranate juice – discrimination between homemade and commercial juices	ICP-OES	Targeted	ANOVA, Tukey’s test	-	Na, Ca	[30]
Authentication of orange juice – Geographical origin discrimination and detection of the adulteration with concentrate	IRMS	Targeted	DA	-	pulp δ^2^H, δ^13^C, δ^15^N, δ^34^S ^87^Sr/^86^Sr	[33]
Authentication of fruit juices - Differentiation according to geographical origin, botanical origin and determination of the addition of sugar	IRMS, SNIF-NMR	Targeted	PCA	Geographical origin prediction ability: 94%	(D/H)_I_, (D/H)_II ethanol_, δ^13^C_ethanol_, δ^13^C_pulp_, δ^13^C_sugars_, δ^18^O_water_	[34]
Authentication of apple juice - Differentiation according to geographical origin and cultivar	IRMS, NMR, ICP-MS, TXRF	Targeted	LDA	Geographical origin prediction ability: 83.9% Cultivar prediction ability: 75.8%	δ^2^H, δ^18^O _water_, δ^15^N, δ^13^C _pulp_, (D/H)_I_, (D/H)_II ethanol_, S, Cl, Fe, Cu, Zn, Sr	[35]
Quality evaluation and authentication of orange juice – Identification of exogenous addition of water & sugar	IRMS, ICP-MS,	Targeted	-	-	δ^2^H, δ^13^C, δ^18^O, Elemental profile	[37]
Authentication of Italian citrus juices – Evaluation of AIJN threshold limits	IRMS, SNIF-NMR	Targeted	ANOVA, PCA	-	(D/H)_I_, (D/H)_II ethanol_, δ^13^C_ethanol_, δ^13^C_pulp_, δ^13^C_sugars_, δ^18^O_water_, δ^15^N_pulp_, δ^18^O_pulp_	[38]
Authentication of fruit juices and wines – Identification of exogenous addition of water	IRMS	Targeted	-	-	δ^18^O_water,_ δ^18^O_ethanol_	[39]
Authentication of lemon juice – Identification of exogenous addition of acidifying agents & sugars	LC-IRMS	Targeted	-	Lowest level of detected adulteration: 10% for citric acid, glycose and fructose	Judgment Ratios: δ^13^C [Citric acid /Glucose], δ^13^C [Citric acid/Fructose], δ^13^C [Glucose/Fructose], δ^13^C [(Tartaric acid + Malic Acid)/2]	[41]
Authentication of lemon juice – Detection of the addition of organic acids & sugars	HPLC-co-IRMS	Targeted	Linear regression	-	δ^13^C of citric acid, glucose and fructose	[42]
Authentication of lemon juice – Differentiation according to geographical origin	ICP-MS	Targeted	LDA, PLS-DA, k-NN, RF, SVM	Correct classification rate: 76% (SVM) 71% (RF)	19 trace elements	[44]
Authentication of grape juice –Classification of organic and conventional juices	ICP-MS	Targeted	PCA, SIMCA	Prediction Ability: PCA: 55% SIMCA: 94% conventional samples, 100% organic	Ba, Ce, La, Mg, P, Pb, Rb, Sn, Ti, Na, Va	[46]
Authentication of grape juice –Differentiation between organic and conventional juices	ICP-MS	Targeted	SVM, MLP, CART,	Correct classification rate: 89.2% (SVM) 71% (CART, MLP)	Na, Sn, P, K, Sm and Nd	[47]
Authentication of orange and apple juices - Detection of the addition of sugars	Ion Chromatography – Carbohydrate Chromatography	Targeted	PCA	Correct classification rate: Apple juice: 94% Orange juice: 80%	K, Na, Mg, Ca, fructose, glucose, saccharose,	[48]
Authentication of pomegranate juice –Detection of juice-to-juice adulteration with peach and grape juice	AAS, AES	Targeted & Untargeted	-	K < 2000 mg L^−^^1^: indicative of adulteration	Ca, Mg, Fe, K	[49]

AAS: Atomic Absorption Spectrometry, AES: Atomic Emission Spectroscopy, ANOVA: Analysis Of Variance, CART: Classification And Regression Tree, DA: Discriminant Analysis, ICP-MS: Inductively coupled plasma mass spectrometry, IRMS: Isotope Ratio Mass Spectrometry, k-NN: k-Nearest Neighbors, LDA: Linear Discriminant Analysis, MLP: Multilayer Perceptron, PLS-DA: Partial Least Squares Discriminant Analysis, RF: Random Forest, SIMCA: Soft Independent Modelling by Class Analogy, SNIF: Site Specific Natural Isotope Fractionation, SVM: Support Vector Machine.

**Table 3:** Applications of spectroscopic techniques in authenticity studies of fruit juices.

Aim of Study	Analytical Technique		Type of Study		Chemometric Approach		Sensitivity & Accuracy	Authenticity Markers	Ref.
Authentication of pomegranate juice – Detection of adulteration with apple, grape - exogenous addition of water	UV-Vis		Untargeted		PCA		Lowest level of detected adulteration: 10%	UV-Vis Spectra	[52]
Analysis of variety, adulteration, quality and ageing of apple juices	UV spectroscopy		Untargeted & Targeted		PCA, PCR, PLSR		Prediction of adulteration: PLSR: Root-mean-square error < 0.7783%, R^2^ > 0.9980	UV spectra Physicochemical parameters	[53]
Authentication of orange juice – Detection of adulteration with grapefruit juice	3D-Front-Face Fluorescence Spectroscopy		Untargeted		ICA		Lowest level of detected adulteration: 1%	3D fluorescence spectra Antiradical Activity, Total Flavonoid Content	[54]
Authentication of apple juice categories (fruit juice vs reconstituted from concentrate)	Synchronous Scanning Fluorescence		Untargeted		PLS-DA, PCA		PLS-DA: Cross-validation error rate: 0.05–0.14 External validation error rate: 0.05–0.44	Total and Specific Synchronous Fluorescence Spectra	[55]
Authentication of bayberry juice – Detection of the adulteration with the addition of water	NIR spectroscopy		Untargeted		PCA, RBFNN		Lowest level of detected adulteration: 10% Recognition Rate: 97.6%	NIR spectra	[56]
Authentication of grape juice – Varietal Differentiation	VIS-NIR & MIR spectroscopy		Untargeted		PCA, LDA, PLS-DA		Correct Classification Rate LDA: 86% (MIR)–80% (NIR) PLS-DA: 86%	VIS-NIR & MIR spectra	[57]
Authentication and differentiation of apple, blueberry, cranberry, concord grape and plum juice	FTIR		Untargeted		PCA, HCA, SIMCA		100% of correct classification	Phenolic-rich fraction spectra	[58]
Authentication of Concord grape juice in different grape juice blends	FTIR		Untargeted		SIMCA, PLSR			FTIR Spectra (phenolic fraction)	[59]
Authentication of pomegranate juice concentrate – Detection of adulteration with grape juice concentrate	FTIR		Untargeted		PCA, PLSR		Lowest level of detected adulteration: 2%	FTIR Spectra 1700–1800 cm^-1^ region, C=O stretching mode.	[60]
Authentication of mango juice - Detection of the addition of sucrose solution	FTIR		Untargeted		PCA, PLSR		Lowest level of detected adulteration: 3–5%	FTIR spectra	[61]
Authentication of orange juice - Detection of the addition of sugar adulterants	FTIR spectroscopy		Targeted		PC-DFA, PLSR		Lowest level of detected adulteration: 0.5–20%	Sucrose, glucose & fructose	[62]
Authentication of orange juice – Detection of the adulteration of freshly squeezed with concentrated orange juices	FTIR-ATR		Untargeted		PCA, LDA		Lowest level of detected adulteration: 10%	FTIR spectra	[63]
Quality Control of fruit juices - Estimation of fruit content of juices	^1^H-NMR		Targeted & Untargeted		PCA, SIMCA		Accuracy: 10%	NMR Spectra	[64]
Authentication of orange juice – Detection of adulteration with clementine juice	^1^H-NMR		Untargeted		PLSR		Lowest Error Rate: 3.47% ofmisclassification	NMR spectra	[65]
Authentication of sour cherry juices – Clones/cultivars discrimination	^1^H-NMR Spectroscopy, Quantitative DescriptiveSensory Analysis,		Untargeted		SIMCA, PCA, PLS-DA		Prediction ability: 82–91.2%	NMR spectra Malic acid, malic acid/glucose ratio	[66]
Authentication of mango juices – Cultivars discrimination		1D and 2D-NMR Spectroscopy		Untargeted		PCA	-	band-selective 1D and 2D NMR arginine, histidine, phenylalanine, glutamine, shikimic acid, trigonelline	[67]
Quality and authentication of orange juice – influence of storage conditions and variety discrimination		^1^H-NMR spectroscopy		Targeted & Untargeted		PCA, PCR, PLSR	-	a-glucose, b-glucose, fructose, ethanol, acids (citric, formic, fumaric, succinic, acetic, lactic, malic, pyruvic)	[68]
Grape juice Quality Control (storage conditions, commercial juice authenticity)		^1^H-NMR spectroscopy		Untargeted		PCA	-	Ethanol, acetate, aminoacids, citric acid, sucrose, sodium benzoate	[69]
Authentication of sweet cherry juice – Geographical Origin Characterization		^1^H-NMR		Untargeted		PCA, LDA, PLS-DA	Prediction ability: 94.9% for PLS-DA 92.3–94.9% for LDA	NMR spectra malate, glucose, fructose, glutamine, succinate	[70]

DA: Discriminant Analysis, FTIR: Fourier Transform Infrared Spectrometer, HCA: Hierarchical Cluster Analysis, ICA: Independent Components Analysis, k-NN: k-Nearest Neighbors, LDA: Linear Discriminant Analysis, NIR: Near-infrared spectroscopy, NMR: Nuclear Magnetic Resonance, PC-DFA: Principal Components-Discriminant Function Analysis, PCR: Principal Component Regression, PLS-DA: Partial Least Squares Discriminant Analysis, PLSR: Partial Least Squares Regression, RBFNN: Radial Basis Function Neural Networks, SIMCA: Soft Independent Modelling by Class Analogy.

**Table 4:** Applications of Thin Layer Chromatography, Capillary Electrophoresis and Gas Chromatography authenticity studies of fruit juices.

Aim of Study	Analytical Technique	Type of Study	Chemometric Approach	Sensitivity & Accuracy	Markers	Ref.
Authentication of berry juice – Determination of anthocyanin and anthocyanidin profile	TLC-densitometry	Targeted	-	-	anthocyanin and anthocyanidin fingerprints	[78]
Authentication of shiikuwasha juice – Detection of adulteration with calamondin juice	TLC, HPLC-UV, GC-MS	Targeted	CDA	Prediction capability: 91.7%	3′,5′-di-C-β-glucopyranosylphloretin, polymethoxylated flavones, γ-terpinene	[79]
Authentication of grape juice – Determination of 11 anthocyanins	TLC-MS	Targeted	-	LOQs: ≤90 ng/zone	cyanidin, delphinidin, malvidin, peonidin, cyanidin-3-glucoside, delphinidin-3-glucoside, malvidin-3-glucoside, peonidin-3-glucoside, pelargonidin, pelargonidin-3- glucoside, malvidin-3.5-diglucoside	[80]
Authentication of pomegranate juice – Detection of adulteration with apple juice	MEKC–LIF	Targeted	-	LOD values: 0.848–3.99 nM	L-Asparagine (Asn)	[86]
Authentication of passion fruit juices	CE	Targeted	PCA	LOD values: 0.01–0.70 mmol/L	proline, glutamate, isoleucine, leucine, phenylalanine, arginine	[87]
Differentiation of fruit juices	CZE–indirect UV detection	Targeted	LDA, MLR	**LDA**: Prediction capability 95% **MLR**: Average Prediction Errors 3.2–3.8%	Sugar concentration ratios (Glu/Fru, Suc/Fru, Sor/Fru, Suc/Glu, Sor/Suc)	[88]
Differentiation of fruit juices	CZE–indirect UV detection	Targeted	LDA	Prediction capability: 95%	Organic acids concentration ratios (fumaric/tartaric, fumaric/citric, fumaric/malic, tartaric/malic, malic/isocitric)	[9]
Authentication of pomegranate juice –Detection of juice-to-juice adulteration with peach and grape juice	GC-MS	Targeted & Untargeted	-	Lowest level of detected adulteration: 10% for peach juice–50% for grape juice	acetic acid, isoamyl butyrate, 1-hexanol, linalool, butyl acetate, isobutyl butyrate, benzyl acetate	[49]
Authentication of shiikuwasha and calamondin juice – Detection of juice-to-juice adulteration	GC-MS	Targeted	-	Lowest level of detected adulteration: 1% of shiikuwasha in calamondin juice	γ-terpinene, linalool	[91]
Authentication of pear juice - Detection of the addition of commercial sweeteners & the addition of pear juice to apple juice	CGC–FID	Targeted	-	Lowest level of detected adulteration: 0.5–3%	Oligosaccharide fingerprint, arbutin	[90]
Authentication of organic lemon juice – Detection of adulteration with industrial lemon juice	GC–FID	Untargeted	Linear regression	-	peak number, total peak area and total peak height	[92]
Authentication of apple juice - Differentiation according to variety and geographical origin	GC–MS	Untargeted	PCA, LDA, SLDA	Overall prediction ability: 89.8–100%	Chromatographic profiles of volatile compounds Butyl acetate, (E)-2-hexenal, Hexyl 2-methylbutanoate, Pentyl acetate	[93]
Authentication of apple juice - Discrimination according to variety	GC–MS, Electronic Nose	Untargeted	PCA, SLDA, SIMCA, PLSR	Overall prediction ability: 94.2% (SLDA), 80–100% (SIMCA)	Volatile compounds profile	[94]
Quality evaluation and authentication of orange juice – Identification of exogenous addition of water & sugar	GC–MS	Untargeted	-	-	Total alcohols, total ketones, total sesquiterpenes/total terpenes without limonene (flavor index)	[37]
Authentication of orange juice – Detection of the addition of flavoring agents	Sensory evaluation, GC-MS	Untargeted	-	-	Dilution index, d-limonene, benzoic acid	[95]
Authentication of orange juices – Variety discrimination – Detection of adulteration	GC-MS, GC-FID	Untargeted	PCA, LDA, ANN, GA	Correct classification rate: 78% (PCA), 100% (LDA-ANN-GA)	MS spectra geranyl acetate, a-pinene, sabinene	[96]

ANN: Artificial Neural Networks, CDA: Canonical Discriminant Analysis, CE: Capillary Electrophoresis, CGC-FID: Capillary Gas Chromatography - Flame Ionization Detection, DA: Discriminant Analysis, GA: Genetic algorithms, HS-SPME: Headspace Solid-Phase Microextraction, LDA: Linear Discriminant Analysis, PCA: Principal Component Analysis, PCR: Principal Component Regression, PLSR: Partial Least Squares Regression, SIMCA: Soft Independent Modelling by Class Analogy, SLDA: Stepwise Linear Discriminant Analysis.

**Table 5:** Applications of Liquid Chromatography to authenticity studies of fruit juices.

Aim of Study	Analytical Technique	Type of Study	ChemometricApproach	Sensitivity & Accuracy	Markers	Ref.
Authentication of red-fruit and vegetable juices - Compositional analyses and characterization	HPLC–UV Fluorescence spectroscopy	Targeted & Untargeted	-	-	Anthocyanins, betacyanins, natural and synthetic pigments, hydroxycinnamic acids, hydroxybenzoic acids, catechins	[97]
Authentication of Citrus fruit juices – characterization, classification of juices and detection of juice-to-juice adulteration	HPLC–DAD–ESI–MS/MS	Targeted & Untargeted	PCA, LDA, PLS-DA, PLSR	**PCA**: 66% of total system variability **LDA**: 100% correct classification **PLS-DA**: 98% correct classification **PLSR**: successful detection of adulteration at 10–70 %	Polyphenolic profiles 17 specific polyphenolic markers	[98]
Authentication of Indian citrus fruit juices - Detection of juice-to-juice adulteration	UPLC-QTOF/MS	Targeted & Untargeted	PCA, SIMCA, OPLS-DA	Lowest level of detected adulteration: Targeted: 2%, untargeted: 1%	didymin, rhoifolin, isorhoifolin, neohesperidin, hesperidin, naringin, narirutin, limonin glucoside, vicenin-2 and relative ratios	[6]
Authentication of lemon juice – Detection of adulteration with bergamot juice	HPLC–DAD, IFU methods	Targeted	CDA	Lowest level of detected adulteration: 1–2%	Naringin, neohesperidin, and neoeriocitrin	[26]
Authentication of pomegranate juice – Characterization and detection of juice-to-juice adulteration with grape, apple, sour cherry juice	HPLC–DAD	Targeted	-	-	Anthocyanins and organic acids	[29]
Authentication of pomegranate juice –Detection of the adulteration with red grape juice	HPLC–DAD–IT–MS/MS	Targeted & Untargeted	-	Lowest level of detected adulteration: 15%	Flavan-3-ols monomers, procyanidin dimers and trimers, anthocyanins	[99]
Authentication of purple grape juice – Detection of adulteration with apple juice	HPLC–DAD, HPLC–RI	Targeted	-	Lowest level of detected adulteration: 5% for phloridzin & 2% for sorbitol	Phlorizin, Sorbitol	[100]
Authentication of apple and pear juice – Detection of juice-to-juice adulteration	HPLC–DAD LC–QTOF/MS NMR	Targeted	-	Lowest level of detected adulteration: 0.6–7%	4-O-p-coumarylquinic acid isorhamnetin-3-O-rutinoside, abscisic acid	[101]
Authentication of apple juice - Differentiation according to cultivar & geographical origin	HPLC–DAD HPLC–UV UPLC–QQQ–MS/MS	Targeted	Mixed-effect ANOVA, LDA	-	Catechin, epicatechin, procyanidin B1, procyanidin B2+B4, quercetin-3-rhamnoside, quercetin-3-glucoside + quercetin-3-galactoside, trans-piceid	[102]
Authentication of apple juice - Differentiation according to variety and geographical origin on the basis of their polyphenol composition	HPLC–DAD UHPLC–Q–Orbitrap	Targeted & Untargeted	ANOVA, PCA, SLDA	Overall prediction ability: 91.2–98.3%	4-caffeoylquinic acid, 4-p-coumaroylquinic acid, (+)-catechin, (−)-epicatechin, procyanidin B1, phloridzin, 3-hydroxyphloretin-2′-O-glucoside, quercetin-3-O-glucoside, quercetin-3-O-arabinoside, quercetin-3-O-rhamnoside	[103]
Authentication of orange juice –Differentiation between fermented & fresh juice	HSCCC, LC-IT-MS^n^, Preparative HPLC, NMR, TLC	Targeted	-	-	Isoamericanol A & Isoamericanoic acid A	[104]
Quality and authentication of orange juice – estimation of juice age and variety discrimination	LC × LC-DAD, APCI-IT-TOF/MS	Targeted	-	-	5,6- and 5,8-epoxycarotenoids	[105]
Authentication of orange juice – Differentiation between fresh and commercial juice	Chiral nano–LC–Q–IT	Targeted	-	LODs: 3–8 ng/mL	D-amino acids	[107]
Characterization and authentication of fruit juices based on amino-acid profiles	HPLC–FLD	Targeted	-	-	Total aminoacids, amino acids profile	[106]
Authentication of pomegranate juice –Detection of juice-to-juice adulteration with peach and grape juice	HPLC-DAD/RI	Targeted & Untargeted	-	-	Τartaric acid, proline, sucrose	[49]
Characterization and authentication of fruit juices - Detection of juice-to-juice adulteration	LC–MS/MS	Targeted	-	Lowest level of detected adulteration: Pomegranate with grape: 5%Pomegranate with apple: 5%	Citric acid, malic acid, tartaric acid, quinic acid, isocitric acid	[108]
Authentication of orange juice – Geographical origin discrimination	HPLC–DAD/RI	Targeted	MANOVA, LDA	-	Organic acids (citric, malic, tartaric, oxalic, fumaric), sugars (fructose, glucose), pH, TA, TDS and EC	[109]
Authentication of pomegranate juice – Development of an International Multidimensional Authenticity Specification Algorithm to detect juice-to-juice adulteration	HPLC–DAD/RI, IRMS, Flame Photometry	Targeted	-		Tartaric acid, sucrose, sorbitol, maltose, potassium	[110]

ANOVA: Analysis of Variance, CDA: Canonical Discriminant Analysis, HSCCC: High-Speed Countercurrent Chromatography, IRMS: Isotope Ratio Mass Spectrometry, LDA: Linear Discriminant Analysis, IT: Linear Ion Trap, MANOVA: Multivariate Analysis of Variance, OPLS-DA: Orthogonal Partial Least Squares Discriminant Analysis, PLS-DA: Partial Least Squares Discriminant Analysis, PLSR: Partial Least Squares Regression, SIMCA: Soft Independent Modelling by Class Analogy, SLDA: Stepwise Linear Discriminant Analysis.

**Table 6:** Applications of untargeted authenticity studies of fruit juices.

Aim of Study	Analytical Technique	Type of Study	Chemometric Approach	Sensitivity & Accuracy	Markers	Ref.
Authentication of pomegranate juices – Detection of dilution or juice-to-juice adulteration	HPLC–DAD–MS/MS	Untargeted	-	-	Anthocyanin concentration, ellagitannin profile, ellagic acid	[113]
Authentication of pomegranate juice –Detection of juice-to-juice adulteration	UPLC–DAD–QTOF/MS	Untargeted	PCA	-	Chlorogenic acid isomers	[5]
Authentication of Indian citrus fruit juices - Detection of juice-to-juice adulteration	UPLC–QTOF/MS	Targeted & Untargeted	PCA, SIMCA, OPLS-DA	Lowest level of detected adulteration: Targeted: 2%, untargeted: 1%	HRMS spectra Didymin, rhoifolin, isorhoifolin, neohesperidin, hesperidin, naringin, narirutin, limonin glucoside, vicenin-2 and relative ratios	[6]
Authentication of pineapple, orange, and grapefruit juices – Detection of juice-to-juice adulteration	UPLC–QTOF/MS	Untargeted	PCA, SIMCA, OPLS-DA	Lowest level of detected adulteration: 1%	HRMS spectra Hesperidine, Narirutin, Naringin, Limonin-17-b-d-glucopyranoside and other 17 biomarkers	[7]
Authentication of citrus juices – Variety and origin discrimination – Detection of adulteration	UPLC–QTOF/MS	Untargeted	PCA, PLS-DA, SIMCA	Lowest level of detected adulteration: 1–5%.	HRMS spectra Narirutin, Limonin-17-b-d-glucopyranoside, Hesperidine, Nomilinin-17-b-d-glucopyranoside, Obacunoic acid-17-b-d-glucopyranoside, Isosakuranentin-7-*O*-rutinoside, Nomilinic acid-17-b-d-glucopyranoside	[112]
Authentication of orange, grapefruit and apple juices – Detection of juice-to-juice adulteration	HPLC–QqQ/LIT MS HPLC–QqTOFMS	Untargeted	PCA, LDA	Lowest level of detected adulteration: 15%	MS spectra Tangeretin, Hesperidin, Naringenin, Limonin, Naringin, *N*-methyl proline, Proline betaine, Tyrosine, Hexose, Sorbitol/mannitol/dulcitol	[111]
Authentication of citrus juices – Discrimination of juices and fruit varieties	LC–QTOF/MS	Untargeted	HCA, PLS-DA, ANOVA	-	HRMS spectra Abscisic acid, limonoids, flavonoids, phenylalanine, ferulic acid hexoside, tryptophan	[114]
Authentication of orange juices – Differentiation of premium organic orange juices	HPLC–HR–MS HS–SPME–GC–MS	Untargeted	PCA, HCA, PLS-DA	PLS-DA: 100% prediction ability	Flavonoids, fatty acids, aldehydes, esters	[115]
Authentication of lemon juice - Detection of the addition of aqueous solution containing citric acid and sucrose	Orbitrap LC–MS/MS	Untargeted	PCA, PLS-DA	Lowest level of detected adulteration: 70%	Total Ion Current Chromatograms (TIC), Total mass spectra (TMS)	[116]

ANOVA: Analysis of Variance, DA: Discriminant Analysis, DSA: Direct Sample Analysis, HCA: Hierarchical Cluster Analysis, LDA: Linear Discriminant Analysis, LRA: Linear Regression Analysis, OPLS-DA: Orthogonal Partial Least Squares Discriminant Analysis, PCR: Principal Component Regression, PLS-DA: Partial Least Squares Discriminant Analysis, PLSR: Partial Least Squares Regression, SIMCA: Soft Independent Modelling by Class Analogy.
